# Eco-Anxiety Profiles, Religiosity, and Sustainable Nutrition in Turkish Adults: A Latent Profile and Network Analysis

**DOI:** 10.3390/nu18030545

**Published:** 2026-02-06

**Authors:** Sedat Arslan, Hande Ongun Yilmaz, Salim Yilmaz

**Affiliations:** 1 Department of Nutrition and Dietetics, Bursa Uludag University, 16059 Bursa, Türkiye; sedatarslan@uludag.edu.tr; 2 Department of Nutrition and Dietetics, Bandirma Onyedi Eylul University, 10250 Balikesir, Türkiye; hyilmaz@bandirma.edu.tr; 3 Department of Healthcare Management, Faculty of Health Sciences, Acibadem Mehmet Ali Aydinlar University, 34752 İstanbul, Türkiye; 4 Department of Healthcare Management, Graduate School of Health Sciences, Acibadem Mehmet Ali Aydinlar University, 34752 İstanbul, Türkiye

**Keywords:** eco-anxiety, religiosity, sustainable nutrition, latent profile analysis, network analysis

## Abstract

**Background:** Eco-anxiety is increasingly viewed as a multidimensional response to the climate crisis, but its links with religiosity and sustainable nutrition behaviors in highly religious settings are unclear. We identified eco-anxiety profiles in Turkish adults; compared religiosity, sustainable nutrition behaviors, and body mass index (BMI) across profiles; and examined the multivariate network connecting these domains. **Methods:** This cross-sectional online survey in Türkiye included 1105 adults (69.3% women; age 25.8 ± 8.4 years; BMI 23.5 ± 4.5 kg/m^2^). Participants completed the Eco-anxiety Scale, Duke University Religion Index, and Behaviors Scale Toward Sustainable Nutrition. Latent profile analysis used four eco-anxiety subscales. Between-profile differences were tested using canonical discriminant analysis and Kruskal–Wallis tests. A Gaussian graphical model estimated with EBICglasso assessed network connectivity. **Results:** Four profiles emerged: High (11.9%), Moderate (54.8%), Affective-dominant (8.3%), and Low (24.9%). Compared with the Low profile, the High profile showed higher sustainable nutrition scores for food preference, seasonal/local nutrition, and food purchasing (all *p* < 0.05); however, effect sizes were small (η^2^_H_ = 0.008–0.014), indicating modest practical differences. BMI did not differ across profiles (*p* = 0.211). In the network, seasonal/local nutrition had the highest strength centrality, whereas BMI was peripheral and weakly connected to other nodes. **Conclusions:** Eco-anxiety was heterogeneous and showed modest associations with sustainable nutrition behaviors at the group level, without differences in BMI. These preliminary findings suggest that eco-anxiety may co-occur with more sustainable food-related choices, generating hypotheses for future replication.

## 1. Introduction

Climate change is widely recognized as one of the greatest threats to planetary and human health in the 21st century, with impacts spanning physical, social, and economic systems [1,2]. Beyond morbidity and mortality, it also imposes substantial psychological burdens, eliciting anxiety, grief, anger, guilt, and hopelessness [3,4]. The American Psychological Association and ecoAmerica describe eco-anxiety as a “chronic fear of environmental doom” rooted in awareness of environmental degradation and concern for present and future generations [3]. Overlapping with “climate anxiety,” eco-anxiety is increasingly observed across populations and is generally viewed as an understandable—though potentially impairing—response to the climate crisis rather than a discrete psychiatric disorder [4,5].

Recent evidence highlights the scale of climate-related distress, especially among younger cohorts. Large multicountry surveys show that most adolescents and young adults report being very or extremely worried about climate change, and many state that these concerns affect daily functioning and future planning [6]. Similar patterns have been reported across high- and middle-income contexts, where climate change is increasingly framed as a public mental health challenge [4,5]. Importantly, eco-anxiety is not uniformly maladaptive; under some conditions it may relate to information-seeking, civic engagement, and pro-environmental behavior [4,7].

Eco-anxiety is a multidimensional construct, spanning affective (e.g., fear, sadness, anger), cognitive (e.g., rumination, catastrophic thinking), behavioral (e.g., avoidance vs. engagement), and functional components [5,8]. Accordingly, dedicated instruments such as the Climate Change Anxiety Scale and multifactor eco-anxiety measures capture emotional–cognitive–behavioral–functional dimensions of climate-related distress [5,8]. Validation work across cultural settings, including recent Turkish adaptations, supports reliable assessment while also indicating that eco-anxiety intensity and symptom expression vary across individuals [9]. Yet, most studies treat eco-anxiety as a continuous dimension, leaving open whether qualitatively distinct profiles combine components differently (e.g., high emotional reactivity with low rumination vs. globally high eco-anxiety).

Alongside these developments, attention has turned to links between climate-related emotions and everyday behaviors, particularly food system-related actions. Food production and consumption substantially contribute to greenhouse gas emissions, biodiversity loss, and resource depletion while also shaping population health [10,11]. In response, the FAO and WHO have advanced sustainable healthy diets—patterns that support health and well-being, exert low environmental pressure, are affordable and culturally acceptable, and promote equity [11,12]. Related behaviors (e.g., reducing food waste, choosing minimally processed and plant-forward foods, preferring local/seasonal products, and environmentally responsible purchasing) are thus key levers for mitigation and noncommunicable disease prevention [10,11,12].

Emerging research suggests positive but often modest and context-dependent associations between climate concern/eco-anxiety and pro-environmental behaviors, including some food-related actions [4,7]. However, studies focusing specifically on sustainable nutrition (rather than general environmental behaviors) remain limited. Even fewer investigations integrate eco-anxiety, sustainable nutrition behaviors, and objective health indicators such as body mass index (BMI) in the same framework. This gap may be particularly important in majority-Muslim contexts, where norms around food, waste, and stewardship could shape how climate-related distress relates to dietary choices.

Religiosity is a potentially influential but understudied factor in this nexus. Religious beliefs and practices inform moral frameworks and daily routines, potentially shaping interpretations of ecological threat and behavioral responses. Meta-analytic and empirical evidence indicates that religiosity can support proenvironmental intentions and behaviors through norms, perceived control, and environmental attitudes, although associations vary across contexts and dimensions of religiosity [13,14]. Recent findings also suggest that religious affiliation and practice intensity may be associated with differences in climate anxiety, possibly buffering or amplifying distress depending on theological narratives (e.g., environmental responsibility, divine control, eschatology) [15]. Yet, the combined interplay of religiosity, eco-anxiety, and sustainable nutrition behaviors remains largely unexamined, especially in highly religious settings.

From a health perspective, obesity and elevated BMI continue to rise globally, including in Türkiye, increasing cardiometabolic burden [16]. More sustainable dietary patterns—often higher in plant foods and lower in ultra-processed, energy-dense products—align with obesity prevention, but empirical links among eco-anxiety, sustainable nutrition, and BMI are still inconsistently characterized. It remains unclear whether higher eco-anxiety relates to more sustainable dietary practices and whether these practices translate into lower BMI or reflect largely independent domains in real-world populations.

Eco-anxiety research increasingly shows that climate-related distress is not monolithic: the same level of “concern” can co-occur with divergent cognitive–affective patterns (e.g., rumination vs. problem-focused engagement) and with markedly different degrees of functional interference [4,5,7,8]. This is consistent with the multidimensional conceptualization of eco-anxiety, in which affective, cognitive, behavioral, and functional components can cluster in different combinations within individuals [5,8]. Such heterogeneity also helps explain why eco-anxiety is not uniformly maladaptive; under some conditions it may coincide with information seeking and civic/pro-environmental engagement, whereas under others it may be associated with impairment and withdrawal [4,7].

Accordingly, an important next step is to move beyond treating eco-anxiety as a single continuous dimension and to identify qualitatively distinct patterns of symptom configuration—i.e., profiles that reflect how emotional reactivity, rumination, engagement/avoidance, and functional impact co-occur in real-world populations [5,8,9]. Person-centered methods such as latent profile analysis (LPA) are well suited to this aim because they explicitly model heterogeneity by identifying subgroups with similar indicator patterns rather than assuming a homogeneous distribution [17]. Establishing such profiles provides a stronger basis for evaluating novelty and generalizability than sample-specific partitioning alone, because profiles can be interpreted against theoretically expected combinations (e.g., low across domains; elevated affective responses with limited functional disruption; high cognitive/functional burden) derived from the multidimensional framework [5,8,17].

Network analysis conceptualizes psychological phenomena as systems of interacting variables and can identify central and bridge components that may inform more targeted interventions [18,19]. Applying this approach to eco-anxiety dimensions, religiosity indicators, sustainable nutrition behaviors, and BMI may clarify how these domains are connected in practice—for example, whether specific religiosity dimensions or sustainability behaviors serve as “bridges” linking eco-anxiety to health-relevant outcomes [18,19,20]. In Türkiye, religious moral frameworks may constitute a culturally salient pathway: Islamic ethics often emphasize stewardship (khalīfa/amanah), moderation, and avoidance of waste (israf), which map onto food-related sustainability behaviors (e.g., reducing waste, avoiding excess consumption). Such norms may buffer impairment-related eco-anxiety by providing meaning while also motivating action by framing sustainability as a moral duty. Accordingly, we expected internalized religiosity to relate to lower eco-anxiety-related functional impairment and to be positively associated with sustainable diet intentions/behaviors, and to be relevant for how climate concern/anxiety translates into food-related sustainability practices in the Turkish context. Against this background, the present study sought to: (i) identify latent profiles of eco-anxiety among adults living in Türkiye using a multidimensional measure; (ii) examine how these profiles differ in terms of sociodemographic characteristics, religiosity, sustainable nutrition behaviors, and BMI; and (iii) model the network structure connecting eco-anxiety dimensions, religiosity indicators, sustainable nutrition behaviors, and BMI. By integrating person-centered (LPA) and network analytic approaches in a large community sample, this study aims to provide a more nuanced understanding of how eco-anxiety is embedded within value systems and everyday dietary practices in a highly religious national context and to generate evidence that may inform value-based and faith-sensitive interventions for sustainable nutrition and climate-related wellbeing.

## 2. Materials and Methods

### 2.1. Study Design and Participants

This research was designed as a cross-sectional, web-based survey study. The target population comprised adults aged 18–64 years residing in Türkiye. Data were collected online between April and June 2025 using an anonymous structured questionnaire created in Google Forms.

The eligibility criteria were as follows: living within the borders of Türkiye, 18–64 years of age, being able to read and understand Turkish, and providing informed consent to participate. Individuals were excluded if they: (i) reported a diagnosed eating disorder, (ii) reported a diagnosed psychiatric disorder, (iii) had impaired oral intake and/or chewing–swallowing difficulties, (iv) were following a special diet under the supervision of a dietitian, (v) had a medically diagnosed food allergy or intolerance, or (vi) were pregnant or breastfeeding, in line with the protocol submitted to the ethics committee. Diagnosed psychiatric disorder and eating disorder were assessed via self-report screening items (yes/no) asking whether the respondent had ever received a professional diagnosis. In this context, “psychiatric disorder” was intended to include common diagnoses such as anxiety and depressive disorders. The “special diet under dietitian supervision” exclusion was applied to reduce heterogeneity introduced by therapeutic or structured dietary regimens that could independently affect eating-related behaviors and related distress.

The questionnaire link was disseminated via social media platforms and messaging groups, and participation was entirely voluntary. Participants could complete the survey only once using the same device and IP address. The final analytical sample comprised 1105 participants after the exclusion of respondents who did not meet the eligibility criteria, duplicate entries, individuals outside the target age range, and cases with missing or implausible anthropometric values. Because a stage-wise attrition log was not retained at the time of data collection, we reconstructed the participant flow from the raw export and the exclusion flags applied during data cleaning. In total, *N* = 1133 survey entries were recorded. We excluded *n* = 1 duplicate entry identified via duplicate/IP/device controls, retaining the first complete submission. We then excluded *n* = 15 entries with missing data on primary eco-anxiety indicators required for LPA and/or key covariates. Next, *n* = 12 entries were removed due to implausible anthropometric values based on predefined plausibility thresholds. The final analytic sample comprised *N* = 1105 participants. Age eligibility was assessed via self-reported age in the survey; respondents outside the 18–64 range were excluded during data cleaning.

### 2.2. Procedure and Data Collection

Upon online questionnaire initiation, participants viewed an information page describing the study aim, scope, and procedures. The page also outlined confidentiality assurances and the right to withdraw at any time without justification. Those who selected the option “I voluntarily agree to participate in this research” were directed to the survey items; those who did not agree were exited from the form. We did not maintain a stage-wise attrition log (i.e., counts excluded at each step) beyond the final cleaned analytical sample; exclusions were applied during data cleaning for duplicates, ineligible age, and missing/implausible anthropometric values.

The data collection form comprised four main sections: Sociodemographic and anthropometric information, eco-anxiety, religiosity, and behavior toward sustainable nutrition.

The completion time was approximately 10–15 min. No monetary or material incentives were provided for participation.

### 2.3. Measures

#### 2.3.1. Sociodemographic and Anthropometric Variables

Sociodemographic characteristics included age (years), sex, marital status, educational level, and employment status, according to the ethics application form.

Anthropometric data (body weight and height) were obtained from self-reporting. BMI was calculated as weight (kg) divided by height squared (m^2^). BMI categories were defined according to WHO cutoffs (underweight <18.5 kg/m^2^, normal weight 18.5–24.9 kg/m^2^, overweight 25.0–29.9 kg/m^2^, and obesity ≥30.0 kg/m^2^).

#### 2.3.2. Eco-Anxiety

Eco-anxiety was measured using the Eco-anxiety Scale, a 13-item instrument assessing four dimensions of eco-anxiety: emotional symptoms, behavioral symptoms, rumination, and anxiety about personal impact. Each item asks how frequently respondents experienced specific eco-anxiety-related thoughts or feelings when thinking about climate change and environmental crises and is rated on a 4-point Likert scale from 1 (“never”) to 4 (“almost always”). Subscale scores and a total score (range 13–52) were calculated by summing the relevant items, with higher scores indicating more severe eco-anxiety. The Turkish Eco-anxiety Scale preserves the original four-factor structure and demonstrates good internal consistency and construct validity [21]. In the present sample, the scale demonstrated excellent internal consistency (Cronbach’s alpha = 0.901; McDonald’s omega = 0.942), with subscale alphas ranging from 0.788 to 0.869. Confirmatory factor analysis supported the four-factor structure (CFI = 0.968, TLI = 0.956, RMSEA = 0.052, SRMR = 0.048). Full psychometric details are provided in Supplementary Material S1.

#### 2.3.3. Religiosity

Religiosity was assessed using the Duke University Religion Index (DUREL), a brief five-item measure that evaluates three major dimensions of religious involvement: organizational religious activity, nonorganizational religious activity, and intrinsic religiosity (items 3–5). Each item is rated on a Likert-type scale, with higher scores indicating higher levels of religious involvement and intrinsic religiosity. DUREL has been widely used in epidemiological studies and shown good reliability and validity across different populations [22].

In this study, item wording was adapted to Turkish in line with previous translations while preserving conceptual meaning. A total religiosity score was computed by summing all items, with higher scores reflecting stronger religiosity. Internal consistency in the present sample was good (Cronbach’s alpha = 0.811; McDonald’s omega = 0.872), with the intrinsic religiosity subscale showing alpha = 0.851. Confirmatory factor analysis indicated excellent fit (CFI = 0.998, TLI = 0.994, RMSEA = 0.033, SRMR = 0.013). See Supplementary Material S1 for details.

#### 2.3.4. Behavior Toward Sustainable Nutrition

Sustainable nutrition-related behaviors were evaluated using the Behaviors Scale Toward Sustainable Nutrition, developed for adults living in Türkiye. The scale contains 29 items and four subdimensions: food preference, food waste reduction, seasonal and local nutrition, and food purchasing. Items are scored on a five-point Likert scale ranging from 1 (“never”) to 5 (“always”). All items are positively keyed; thus, higher scores indicate more favorable behaviors toward sustainable nutrition. For each participant, a total sustainable nutrition behavior score was obtained by summing all items (possible range 29–145), and subscale scores were computed by summing items within each subdimension (food preference: 6 items, possible range 6–30; food waste reduction: 9 items, possible range 9–45; seasonal and local nutrition: 8 items, possible range 8–40; food purchasing: 6 items, possible range 6–30) [23]. In this study sample, the scale exhibited excellent internal consistency (Cronbach’s alpha = 0.958; McDonald’s omega = 0.968), with subscale alphas ranging from 0.852 to 0.914. Confirmatory factor analysis confirmed the four-factor structure with acceptable fit (CFI = 0.919, TLI = 0.909, RMSEA = 0.053, SRMR = 0.056). Detailed psychometric evaluation including model modifications is presented in Supplementary Material S1.

### 2.4. Ethical Considerations

The study protocol was reviewed and approved by the Non-Interventional Research Ethics Committee of Bandirma Onyedi Eylül University (protocol code 2024-234, 21 October 2024). The research followed the Declaration of Helsinki principles and national regulations on research involving human participants.

### 2.5. Analysis Flow

**Figure 1:**
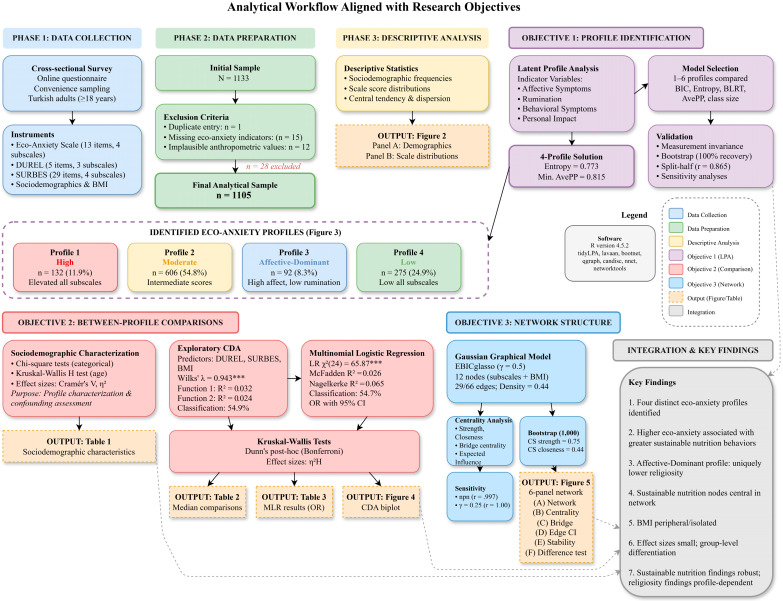
Analytical flowchart and research objectives. The workflow progresses from data collection and preparation through latent profile analysis (Objective 1), between-profile comparisons using canonical discriminant analysis and multinomial logistic regression (Objective 2), and network analysis using Gaussian graphical modeling (Objective 3). Color coding indicates the analytical phase, with outputs (tables and figures) shown at their respective stages. *** *p* < 0.001.

The analytical workflow of this study comprised eight sequential phases designed to address the three research objectives (Figure 1).

Objective 1 (Profile Identification): Following data collection through an online cross-sectional survey and data preparation procedures that yielded a final sample of 1105 participants, descriptive analyses characterized the sociodemographic and psychometric properties of the sample. LPA was then employed to identify distinct subgroups based on eco-anxiety symptom patterns, resulting in a four-profile solution. Measurement invariance testing and bootstrap stability analyses validated the robustness of the identified profiles.

Objective 2 (Between-Profile Comparisons): Sociodemographic characteristics were compared across profiles to characterize their composition and assess potential confounding. Exploratory CDA and multinomial logistic regression examined whether religiosity, sustainable nutrition behaviors, and BMI differed across the identified profiles, with follow-up nonparametric Kruskal–Wallis tests and Dunn’s post hoc comparisons corroborating the findings.

Objective 3 (Network Structure): Network analysis using Gaussian graphical models elucidated the structural connectivity patterns among eco-anxiety, religiosity, sustainable nutrition behaviors, and BMI at the subscale level, with bootstrap procedures ensuring parameter stability and accuracy.

### 2.6. Statistical Analyses

All statistical analyses were performed using R software version 4.5.2. Following descriptive characterization of the sample through frequencies, percentages, means, and standard deviations, LPA was conducted using the tidyLPA package [24] to identify homogeneous subgroups based on the four eco-anxiety subscales, with model selection guided by BIC, entropy, BLRT, average posterior probabilities, and minimum class size criteria. Models specifying one through six profiles were compared; detailed fit indices including AIC, BIC, sample-adjusted BIC, entropy, and classification diagnostics are presented in Supplementary Material S2 (Tables S5 and S6).

Prior to between-group comparisons, measurement invariance of the Eco-Anxiety Scale was tested across gender (male vs. female) and age groups (18–24 vs. 25+ years) using multigroup confirmatory factor analysis with robust maximum likelihood estimation. Configural, metric, scalar, and strict invariance models were compared using changes in CFI (|ΔCFI| < 0.010) and RMSEA (|ΔRMSEA| < 0.015) as recommended by Chen [25]. Profile stability was evaluated through bootstrap resampling (1000 iterations) and split-half cross-validation. Sensitivity analyses were also conducted excluding profiles with moderate replication to assess the robustness of between-profile differences.

Sociodemographic characteristics were then compared across the identified profiles using chi-square tests for categorical variables and Kruskal–Wallis H tests for continuous variables, with effect sizes reported as Cramér’s V and eta-squared, respectively. To examine whether religiosity, sustainable nutrition behaviors, and BMI could discriminate among profiles, exploratory canonical discriminant analysis (CDA) was performed using the candisc package [26], with structure coefficients interpreted to identify the substantive meaning of significant functions, followed by Kruskal–Wallis tests with Dunn’s post hoc comparisons (Bonferroni-corrected) to corroborate findings. Given potential violations of CDA’s homogeneous covariance assumption (assessed via Box’s M test) and to provide interpretable effect sizes, multinomial logistic regression was conducted as a complementary analysis to examine associations between profile membership and standardized predictor variables, with odds ratios and 95% confidence intervals reported.

Network analysis using Gaussian graphical models (GGMs) estimated via the EBICglasso algorithm (γ = 0.5) was conducted with the qgraph [27] and networktools [28] packages to examine the structural relationships among all study variables at the subscale level. This analysis was performed on complete cases (*n* = 1105), as all scale items were mandatory and cases with missing anthropometric data had been excluded during data preparation. Although GGM assumes approximate multivariate normality, sensitivity analysis using nonparanormal transformation confirmed that all network parameters remained virtually identical (edge weights r = 0.997, centrality indices r > 0.97), indicating robustness to distributional assumptions. Additional sensitivity analysis comparing γ = 0.50 versus γ = 0.25 regularization parameters revealed identical network structures (all r = 1.00), confirming robustness to the choice of tuning parameter. Centrality indices, bridge centrality, and bootstrap stability analyses (1000 iterations) were performed using the bootnet package [20] to ensure network parameter accuracy and replicability. Cross-construct edges were interpreted as weak partial associations given their small magnitudes (r = 0.05–0.09), and causal or mechanistic interpretations were avoided. The significance level was set at *p* < 0.05 for all analyses, and effect sizes were reported alongside statistical significance to evaluate practical importance.

## 3. Results

The final analytical sample comprised 1105 participants following data cleaning procedures, which included the exclusion of individuals under 18 years of age and cases with missing or implausible anthropometric data.

**Figure 2:**
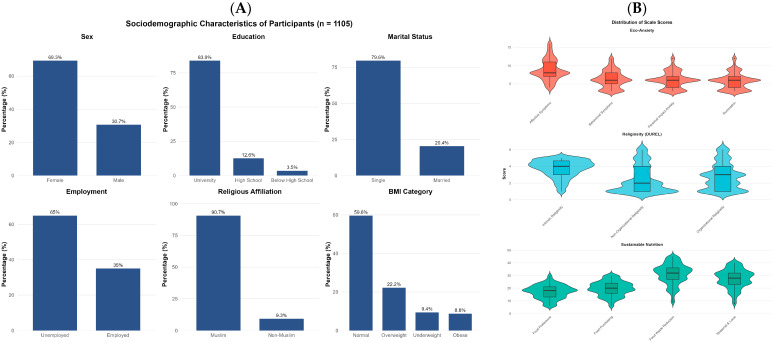
Descriptive characteristics of the study sample. (**A**) displays the sociodemographic distribution of participants with percentages. (**B**) presents the distribution of scale scores across Eco-anxiety (4 subscales), Religiosity (DUREL; 3 subscales), and Sustainable Nutrition (4 subscales) using violin plots with embedded box plots showing median and interquartile range. Eco-anxiety subscale scores: Affective Symptoms (M = 8.12, SD = 2.89), Rumination (M = 6.45, SD = 2.67), Behavioral Symptoms (M = 7.21, SD = 2.54), Personal Impact (M = 5.23, SD = 2.18). *N* = 1105.

The sample was predominantly female (69.3%), single (79.6%), and university-educated (83.9%). The mean age was 25.75 years (SD = 8.41), and the mean BMI was 23.52 kg/m^2^ (SD = 4.50), with most participants falling within the normal weight category (59.6%). Regarding religious affiliation, the vast majority identified as Muslim (90.7%). Employment status indicated that 65.0% of participants were unemployed, which is consistent with the young, student-dominated sample composition (Figure 2A).

Examination of the scale scores revealed moderate levels of eco-anxiety (M = 27.01, SD = 7.49, possible range: 13–52), with affective symptoms representing the highest scoring subscale. Religiosity scores demonstrated considerable variability, with intrinsic religiosity (M = 3.74, SD = 1.04) being notably higher than organizational (M = 2.76, SD = 1.55) and non-organizational religiosity (M = 2.42, SD = 1.63). Sustainable nutrition behaviors were moderately high (M = 95.50, SD = 23.34, possible range: 29–145), with food waste reduction behaviors scoring highest among the subscales (Figure 2B).

To identify distinct subgroups of individuals based on their eco-anxiety profiles, LPA was conducted using the four eco-anxiety subscales as indicator variables. Models specifying one through six latent profiles were estimated and compared using multiple fit indices, including BIC, entropy, minimum posterior probability, smallest class proportion, and BLRT. The four-profile solution was selected as optimal based on the balance of statistical fit and interpretability: although the six-profile model yielded a lower BIC, its smallest class comprised only 3.4% of the sample, which was considered insufficient for meaningful between-group comparisons. The four-profile solution demonstrated acceptable entropy (0.773) and adequate minimum posterior probability (0.716), and all profiles exceeded the recommended 5% threshold for class size.

Profile-specific means with standard errors and standardized z-scores are provided in Supplementary Material S2 (Table S6). The z-scores clearly illustrate profile distinctiveness: the High profile exhibited uniformly elevated scores across all subscales (z = 1.18 to 1.60), the Low profile showed consistently below-average scores (z = −0.72 to −0.96), and the Moderate profile clustered near the sample mean (z = −0.14 to 0.15). The Affective-Dominant profile displayed a distinctive pattern with elevated affective (z = 1.37) and behavioral symptoms (z = 0.91) but notably lower rumination (z = −0.27), validating its characterization as emotionally reactive but non-ruminative eco-anxiety.

Model selection also involved the comparison of variance–covariance structures. Alternative specifications, including varying variances and nonzero covariances, either failed to converge or yielded unacceptable fit indices (e.g., entropy = 0.300). Classification quality was further validated through the examination of average posterior probabilities for each profile, which ranged from 0.815 to 0.897, all exceeding the recommended threshold of 0.70 and indicating high classification precision. Figure 2 displays the score distributions for each profile across the four eco-anxiety dimensions, revealing a clear separation between profiles and distinct distributional shapes that validate the LPA classification.

**Figure 3:**
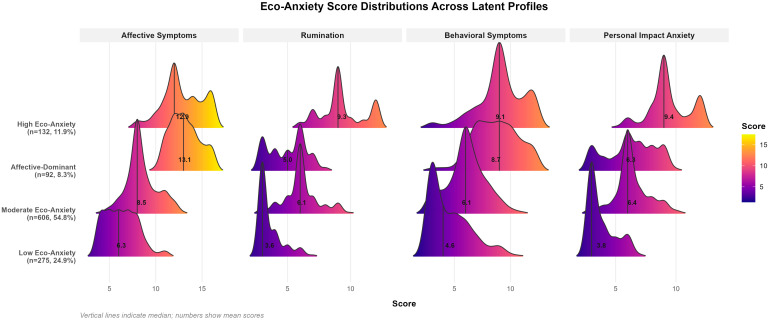
Eco-anxiety subscale scores in four latent profiles determined by LPA. Ridgeline density plots display score distributions for each eco-anxiety subscale across profiles. Profile-specific sample sizes and percentages are shown on the y-axis. Vertical lines indicate median values; numbers represent mean scores. Color gradient reflects score magnitude (yellow = higher, purple = lower). Total *n* = 1105.

The four identified profiles were labeled based on their characteristic patterns across the eco-anxiety subscales. Profile 1 (high eco-anxiety; *n* = 132, 11.9%) exhibited uniformly elevated scores across all four subscales, indicating pervasive eco-anxiety symptomatology. Profile 2 (moderate eco-anxiety; *n* = 606, 54.8%) represented most participants and displayed intermediate scores across all dimensions. Profile 3 (affective-dominant; *n* = 92, 8.3%) demonstrated a distinctive pattern characterized by elevated affective and behavioral symptoms but notably lower rumination, suggesting emotionally reactive but non-ruminative eco-anxiety. Profile 4 (low eco-anxiety; *n* = 275, 24.9%) exhibited consistently low scores across all subscales (Figure 3).

Prior to addressing the second study objective (between-profile comparisons), measurement invariance of the Eco-Anxiety Scale was tested to ensure that the instrument measured the same constructs equivalently across gender and age groups. For gender, fit index changes across invariance levels were minimal: configural to metric (ΔCFI = −0.001, ΔRMSEA = −0.002, ΔSRMR = 0.001), metric to scalar (ΔCFI = −0.006, ΔRMSEA = 0.002, ΔSRMR = 0.001), and scalar to strict (ΔCFI = 0.001, ΔRMSEA = −0.003, ΔSRMR = 0.000). Similarly, for age groups, all changes remained within acceptable thresholds: configural to metric (ΔCFI = 0.000, ΔRMSEA = −0.002, ΔSRMR = 0.001), metric to scalar (ΔCFI = −0.002, ΔRMSEA = 0.000, ΔSRMR = 0.000), and scalar to strict (ΔCFI = −0.002, ΔRMSEA = −0.002, ΔSRMR = 0.001). All values fell within recommended criteria (|ΔCFI| < 0.010, |ΔRMSEA| < 0.015, |ΔSRMR| < 0.030), supporting the validity of subsequent between-group profile comparisons (Tables S7 and S8 in Supplementary Material S2). Bootstrap analysis demonstrated that the four-profile solution was recovered in 100% of 1000 resamples. Split-half cross-validation revealed high pattern correlations between independently estimated profile centroids across subsamples (mean r = 0.865), with three profiles showing excellent replication (r = 0.929–0.985) and the Affective-Dominant profile showing moderate replication (r = 0.585), consistent with its smaller size (Table S9) and distinctive configuration. Given this reduced stability, interpretations involving the Affective-Dominant profile should be considered preliminary; sensitivity analyses examining the robustness of key findings when excluding this profile are presented in Supplementary Material S3.

To characterize the demographic composition of each profile and assess whether profiles differed substantially in sociodemographic features that might confound subsequent between-profile comparisons, Table 1 presents the sociodemographic characteristics of the four eco-anxiety profiles. Chi-square tests and Kruskal–Wallis analysis revealed statistically significant but practically small differences across profiles for age, age group, and gender. Participants in the High eco-anxiety profile were slightly younger (Median = 22, Q1–Q3: 21–24) than those in the Low eco-anxiety profile (Median = 23, Q1–Q3: 21–28), with a higher proportion of young adults (18–24 years) in the High eco-anxiety profile (76.5%) compared to the Low eco-anxiety profile (62.5%). Gender distribution also differed significantly across profiles (χ^2^ = 19.70, *p* < 0.001, V = 0.131), with females overrepresented in the High eco-anxiety (75.0%) and Affective-Dominant (80.4%) profiles, whereas males were more prevalent in the Low eco-anxiety profile (40.0%). Marital status, education level, and religious affiliation did not differ significantly across profiles (all *p* > 0.05). Effect sizes were uniformly small across all comparisons (Cramér’s V range: 0.055–0.131; η^2^ = 0.006), indicating that while statistically significant, the practical magnitude of sociodemographic differences between profiles was modest, and thus unlikely to account for the between-profile differences in religiosity, sustainable nutrition behaviors, and BMI examined subsequently.

Addressing the second study objective, exploratory canonical discriminant analysis (CDA) was conducted to examine whether religiosity (DUREL subscales), sustainable nutrition behaviors (SURBES subscales), and BMI could discriminate among the four eco-anxiety profiles identified through LPA (Figure 3). Prior to the analysis, assumptions were evaluated. Box’s M test indicated a violation of homogeneity of covariance matrices (χ^2^ = 180.57, *p* < 0.001), and Mardia’s test revealed a departure from multivariate normality (skewness *p* < 0.001; kurtosis *p* < 0.001). However, CDA is robust to these violations with large sample sizes (*n* = 1105), and nonparametric Kruskal–Wallis tests were employed as follow-up analyses to corroborate the findings. Examination of the correlation matrix revealed no problematic multicollinearity among predictors (all r < 0.80). The overall model was statistically significant (Wilks’ λ = 0.943, F_(24,3173.5)_ = 2.68, *p* < 0.001), indicating that the predictor variables collectively differentiated among profiles. Two canonical functions emerged as significant: Function 1 (canonical R^2^ = 0.032, *p* < 0.001) and Function 2 (canonical R^2^ = 0.024, *p* = 0.014), together accounting for 97.1% of the between-group variance. Table 2 summarizes the descriptive statistics for each predictor variable across the four eco-anxiety profiles. Follow-up Kruskal–Wallis tests were conducted to corroborate the discriminant findings and identify which variables demonstrated significant between-profile differences.

Despite statistical significance, the model’s practical discriminatory power was limited. According to canonical R^2^ values, religiosity, sustainable nutrition behaviors, and BMI collectively explained only a small proportion of the variance in profile membership. Classification accuracy (54.9%) essentially equaled the largest group’s base rate (54.8%), indicating that the predictor variables, while differentiating profiles at the group level, provided no incremental predictive utility at the individual level. To address potential violations of CDA’s homogeneous covariance assumption and provide interpretable effect sizes, multinomial logistic regression was conducted as a complementary analysis. Effect sizes for significant Kruskal–Wallis tests were uniformly small ($\eta_{H}^{2}$ range: 0.008–0.014), consistent with typical findings in psychological research. Post hoc Dunn comparisons (Bonferroni-corrected) revealed significant between-profile differences for five of the eight predictor variables. Among sustainable nutrition behaviors, food purchasing (χ^2^ = 18.68, *p* < 0.001, $\eta_{H}^{2}$ = 0.014) showed the clearest differentiation, with the high eco-anxiety profile scoring significantly higher than all other profiles (all *p* < 0.05). Food preference (χ^2^ = 17.22, *p* < 0.001, $\eta_{H}^{2}$ = 0.013) and seasonal and local nutrition (χ^2^ = 11.62, *p* = 0.009, $\eta_{H}^{2}$ = 0.008) followed a similar pattern, with pairwise comparisons revealing significant differences primarily between the high and low profiles. This gradient suggests that individuals experiencing greater eco-anxiety tend to engage more actively in sustainable food-related behaviors. In contrast, food waste reduction behaviors did not differ significantly across profiles (*p* = 0.144). Regarding religiosity, organizational religiosity (χ^2^ = 11.34, *p* = 0.010, $\eta_{H}^{2}$ = 0.008) and intrinsic religiosity (χ^2^ = 12.52, *p* = 0.006, $\eta_{H}^{2}$ = 0.009) differed significantly across profiles. However, this pattern contrasted with that observed for sustainable nutrition behaviors. For intrinsic religiosity, post hoc comparisons revealed that the affective-dominant profile exhibited significantly lower scores than all other profiles (all *p* < 0.05), whereas the high, moderate, and low profiles did not differ from each other. This distinctive pattern tentatively suggests that emotionally reactive but non-ruminative eco-anxiety may be associated with lower internalized religious commitment. However, given the moderate replication stability of the Affective-Dominant profile (r = 0.585), these religiosity-related findings should be interpreted with caution. Sensitivity analyses excluding this profile (Supplementary Material S3) revealed that organizational religiosity (*p* = 0.065) and intrinsic religiosity (*p* = 0.902) differences became non-significant, whereas sustainable nutrition associations remained robust (all *p* < 0.05), indicating that religiosity-related findings are substantially dependent on this less stable profile. Nonorganizational religiosity and BMI did not significantly differentiate among the eco-anxiety profiles (Table 2).

Multinomial logistic regression predicting profile membership (reference category: Low eco-anxiety) from standardized religiosity subscales, sustainable nutrition subscales, and BMI yielded a statistically significant overall model (LR χ^2^(24) = 65.87, *p* < 0.001), though effect size was modest (McFadden R^2^ = 0.026; Nagelkerke R^2^ = 0.065). Consistent with CDA findings, sustainable nutrition behaviors emerged as the primary discriminators of profile membership (Table 3).

Food preference significantly predicted increased odds of membership in the Moderate profile (OR = 1.25, 95% CI [1.02, 1.52], *p* = 0.030) and High profile (OR = 1.58, 95% CI [1.17, 2.14], *p* = 0.003) relative to the Low profile. Food purchasing also predicted High profile membership (OR = 1.47, 95% CI [1.02, 2.12], *p* = 0.041). For the Affective-Dominant profile, seasonal and local nutrition was the sole significant predictor (OR = 1.60, 95% CI [1.04, 2.47], *p* = 0.032). Notably, neither religiosity subscales nor BMI significantly predicted profile membership in any comparison, though organizational religiosity approached significance for High (OR = 0.77, *p* = 0.053) and Affective-Dominant (OR = 0.74, *p* = 0.061) profiles, suggesting a trend toward lower organizational religious participation among higher eco-anxiety groups. Classification accuracy from multinomial logistic regression (54.7%) mirrored that of CDA, confirming that while these variables differentiate profiles at the group level, individual-level prediction remains limited (Table 3).

**Figure 4:**
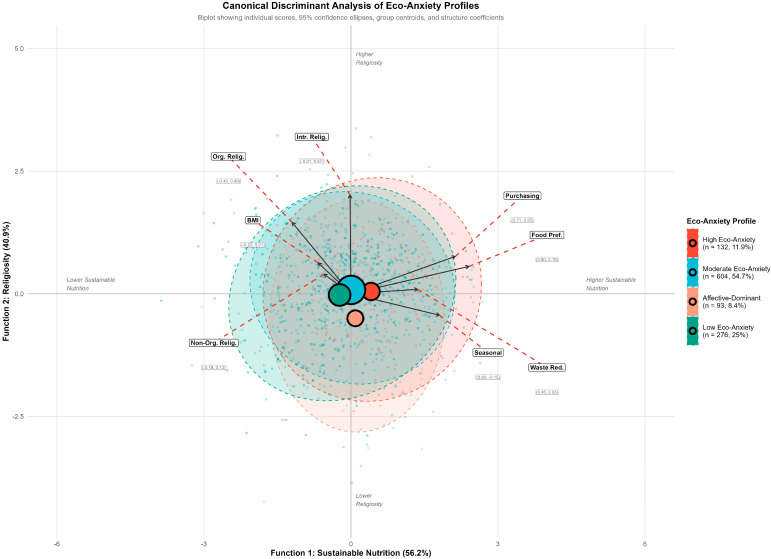
Canonical discriminant analysis biplot displaying the distribution of eco-anxiety profiles in discriminant space. Individual data points represent participants colored by profile membership. Large circles indicate group centroids, with circle size proportional to profile sample size. Dashed ellipses represent 95% confidence regions for each profile. Black arrows originating from the origin show structure coefficients for predictor variables, with coefficient values displayed in parentheses (Function 1, Function 2). Red dashed lines connect arrow tips to displaced labels for readability. Function 1 (56.2% of variance) primarily reflects sustainable nutrition behaviors (Food Preference = 0.80, Purchasing = 0.71, Seasonal = 0.60); Function 2 (40.9% of variance) primarily reflects religiosity dimensions (Intrinsic Religiosity = 0.67, Organizational Religiosity = 0.49). The axis polarity of Function 2 was inverted for intuitive interpretation, such that higher values correspond to higher religiosity. Total *n* = 1105; Wilks’ Lambda = 0.943, *p* < 0.001.

Returning to the CDA results, the structure coefficients revealed the substantive meaning of the discriminant functions. Function 1, accounting for 56.2% of the discriminant variance, was primarily defined by sustainable nutrition behaviors, with food preference ($r_{s}$ = 0.80), food purchasing ($r_{s}$ = 0.71), seasonal and local nutrition ($r_{s}$ = 0.60), and food waste reduction ($r_{s}$ = 0.45) all loading positively. Organizational religiosity negatively impacted this function ($r_{s}$ = −0.40). Thus, Function 1 can be interpreted as a “Sustainable Nutrition” dimension, with higher scores indicating greater engagement in environmentally conscious food behaviors and somewhat lower organizational religious participation. Function 2, accounting for 40.9% of the variance, was predominantly characterized by intrinsic religiosity ($r_{s}$ = 0.67) and organizational religiosity ($r_{s}$ = 0.49), representing a “Religiosity” dimension, where higher scores indicate greater religious commitment. The axis polarity of Function 2 was inverted from the original extraction for intuitive interpretation. Figure 4 illustrates how the structure coefficients clarified the substantive meaning of the two significant canonical functions.

The positioning of group centroids in the discriminant space provides further insight into profile characteristics. The high eco-anxiety profile occupied the positive end of Function 1 (centroid = 0.41), indicating elevated sustainable nutrition behaviors compared to the other profiles. Conversely, the low eco-anxiety profile was positioned at the negative end (centroid = −0.22), reflecting lower engagement in sustainable food practices. Most notably, the affective-dominant profile was distinctly positioned on Function 2 (centroid = −0.50), indicating markedly lower religiosity relative to the other three profiles, which clustered near zero on this dimension. This spatial configuration corroborates the Kruskal-Wallis findings and visually demonstrates the dual nature of profile differentiation: eco-anxiety severity relates primarily to sustainable nutrition behaviors, whereas the affective-dominant profile is distinguished by its lower religiosity rather than its sustainable nutrition patterns (Figure 4).

Collectively, both CDA and multinomial logistic regression indicate that eco-anxiety profiles exhibit modest but theoretically meaningful associations with sustainable nutrition behaviors, with limited contribution from religiosity and BMI. The convergent findings across methods—despite different distributional assumptions—strengthen confidence in these associations, while the equivalent classification accuracy (≈55%) across both approaches confirms that individual-level prediction remains limited. Network analysis was conducted to further elucidate the structural connectivity patterns among eco-anxiety, religiosity, sustainable nutrition, and BMI beyond group-level comparisons.

**Figure 5:**
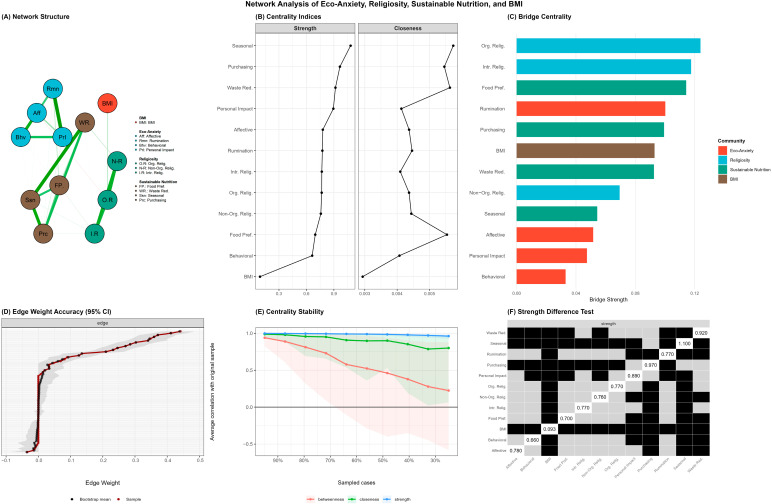
Network analysis of eco-anxiety, religiosity, sustainable nutrition behaviors, and BMI. (**A**) Regularized partial correlation network estimated using EBICglasso. Node colors represent construct communities: eco-anxiety (red), religiosity (blue), sustainable nutrition (green), and BMI (brown). Edge thickness reflects the magnitude of partial correlations; blue edges indicate positive associations, and red edges indicate negative associations. (**B**) Centrality indices (strength and closeness) for all network nodes; values are standardized z-scores. (**C**) Bridge centrality indicating nodes that connect different construct communities. (**D**) Bootstrap 95% confidence intervals for edge weights based on 1000 nonparametric bootstrap samples. (**E**) Centrality stability analysis showing average correlations between original and subset-based centrality indices across varying proportions of sampled cases; CS-coefficients: strength = 0.75 (excellent), closeness = 0.44 (acceptable), betweenness = 0.05 (unstable). (**F**) Bootstrap difference test for strength centrality; black squares indicate statistically significant differences between nodes (α = 0.05).

Addressing the third study objective, a Gaussian graphical model was estimated using the EBICglasso algorithm with LASSO regularization (tuning parameter γ = 0.5) to examine the structural relationships among eco-anxiety, religiosity, sustainable nutrition behaviors, and BMI beyond profile-based comparisons. The network included 12 nodes representing the four eco-anxiety subscales, three religiosity subscales, four sustainable nutrition subscales, and BMI. The resulting network contained 29 nonzero edges out of 66 possible connections (density = 0.44), indicating a moderately sparse structure after regularization (Figure 5A).

The strongest edges emerged within the construct communities rather than between them. Within eco-anxiety, the Personal Impact–Rumination connection exhibited the highest weight in the entire network (r = 0.44), followed by Affective–Behavioral (r = 0.36) and Behavioral–Personal Impact (r = 0.27). Within sustainable nutrition behaviors, seasonal and local nutrition served as a hub connecting strongly to waste reduction (r = 0.41), purchasing (r = 0.37), and Food Preference (r = 0.25). The religiosity subscales formed a tightly interconnected cluster, with Intrinsic–Non-Organizational (r = 0.35), Non-Organizational–Organizational (r = 0.34), and intrinsic–organizational (r = 0.30) connections. Notably, BMI appeared largely isolated from the network, exhibiting only a weak connection with organizational religiosity (r = 0.09). Cross-construct edges were considerably weaker than within-construct connections. Weak partial associations between eco-anxiety and sustainable nutrition were observed, including Rumination–Food Preference (r = 0.08) and Personal Impact–Purchasing (r = 0.05). Similarly, a small partial association linked Non-Organizational Religiosity to Waste Reduction (r = 0.07). These weak cross-construct associations should be interpreted with caution given their small effect sizes; while statistically reliable with the present sample size, their practical significance is limited (Figure 5).

Centrality analysis revealed that sustainable nutrition nodes occupied the most central positions in the network (Figure 5B). Seasonal and local nutrition exhibited the highest strength centrality (z = 1.32), followed by purchasing (z = 0.83), Waste Reduction (z = 0.63), and personal impact anxiety (z = 0.54). BMI demonstrated the lowest centrality across all indices, confirming its peripheral position in the network structure.

Bridge centrality analysis identified nodes serving as connectors between construct communities (Figure 5C). Organizational Religiosity (bridge strength = 0.124) and Intrinsic Religiosity (bridge strength = 0.118) emerged as primary bridges from the religiosity cluster, connecting to BMI and sustainable nutrition behaviors, respectively. Food Preference (bridge strength = 0.114) served as the main bridge from sustainable nutrition to eco-anxiety via its connection with Rumination. Within eco-anxiety, Rumination (bridge strength = 0.100) functioned as the primary bridge to other constructs, consistent with its role as a cognitive process linking emotional experiences to behavioral outcomes.

Network stability and accuracy were assessed using bootstrap procedures with 1000 iterations (Figure 5D–F). The correlation stability coefficient for strength centrality was 0.75, substantially exceeding the recommended threshold of 0.50 and indicating excellent stability. Closeness centrality demonstrated acceptable stability (CS = 0.44, above the minimum threshold of 0.25). Betweenness centrality was unstable (CS = 0.05) and therefore excluded from interpretation, a common finding in psychological network studies. Edge weight accuracy analysis revealed that the strongest edges were estimated with adequate precision, with bootstrap confidence intervals for the top edges not overlapping zero. The strength difference test confirmed that Seasonal and Local Nutrition had significantly higher strength centrality than most other nodes, whereas BMI had significantly lower strength than all other nodes.

Sensitivity analyses confirmed the robustness of network findings. Re-estimation using nonparanormal transformation to relax normality assumptions yielded near-identical results (edge weight r = 0.997, bridge centrality r = 0.977). Similarly, alternative regularization (γ = 0.25 vs. 0.50) produced identical network structures (all r = 1.00), with the same nodes identified as key bridges across specifications. To facilitate interpretation, we assigned descriptive labels to the four latent profiles based on theoretically expected configurations implied by the multidimensional framework of eco-anxiety (affective, cognitive, behavioral, and functional components) and the logic of person-centered modeling [5,8,17]. Specifically, the profiles were labeled as Profile 1 (globally low), Profile 2 (moderate/engaged), Profile 3 (affective-dominant), and Profile 4 (high cognitive–functional burden), reflecting differences not only in overall severity but also in the patterning of emotional reactivity, rumination/engagement, and functional impact across individuals [5,8,17].

## 4. Discussion

This study used a person-centered and network-based approach to examine how eco-anxiety, religiosity, sustainable nutrition behaviors, and BMI co-occur in a large community sample. Four distinct eco-anxiety profiles were identified, ranging from globally low symptoms to a high eco-anxiety subgroup, together with an affective-dominant pattern. Higher eco-anxiety was accompanied by more favorable sustainable nutrition behaviors, whereas lower eco-anxiety coincided with weaker engagement in sustainable eating. Network findings further suggest that sustainable nutrition occupies a structurally central position in the system, while BMI remains largely peripheral, and that specific eco-anxiety and religiosity components act as bridges between emotional experiences and everyday food-related practices. Overall, the results indicate that eco-anxiety is a heterogeneous construct that can be linked to psychological burden and potentially adaptive engagement in sustainable behaviors.

Our four-profile solution supports the heterogeneity view that eco-anxiety comprises different configurations of affective, cognitive, behavioral, and functional components rather than a single continuum [4,5,7,8]. The observed separation between profiles with stronger affective reactivity versus those with higher cognitive/functional burden is consistent with the notion that climate-related distress can be coupled with engagement in some cases and impairment/withdrawal in others [4,7]. By linking profiles to sustainable nutrition behaviors, religiosity, and BMI, we extend this framework into a climate-relevant behavioral domain and a culturally salient value system in Türkiye [10,11,12,13,14,15].

The positive association between eco-anxiety and sustainable eating in our sample converges with recent international and Turkish studies linking higher climate-related worry to greener consumption, sustainable food choices, and adherence to plant-forward dietary patterns [29,30,31,32,33,34,35,36]. For example, Mathers-Jones and Todd reported that higher eco-anxiety predicted greater engagement in daily proenvironmental behaviors among young adults, although at the cost of increased internalizing symptoms [29]. Gkargkavouzi similarly concluded that eco-anxiety can foster sustainable consumption when individuals perceive sufficient efficacy and behavioral options [30]. In the domain of food, Kabasakal-Cetin showed that eco-anxiety was positively associated with sustainable eating and higher EAT-Lancet diet scores in university students [33], while Memiç-İnan and Şarahman Kahraman observed that concern for ecological health co-occurred with healthier and more sustainable dietary patterns in young adults [34]. Corroborating these findings, our high eco-anxiety profile showed the most favorable scores across sustainable nutrition subscales, suggesting that, at least in this relatively young and educated sample, eco-anxiety may represent a “concerned, engaged” phenotype rather than a paralyzed or avoidant one.

The present results also resonate with Turkish studies that directly examine the link between eco-anxiety and sustainable food consumption. Memiç-İnan and Şarahman Kahraman reported that adults with higher eco-anxiety scores displayed more sustainable purchasing and eating preferences [35], and Özkara found that eco-anxiety predicted sustainable consumption behaviors through increased ecological footprint awareness [36]. These findings align closely with our network results, where sustainable nutrition nodes occupied central positions and eco-anxiety was connected to them through specific cognitive and behavioral bridges rather than uniformly across all items. Converging evidence suggests that eco-anxiety, when accompanied by adequate knowledge, perceived behavioral control, and supportive social norms, can be channeled into sustainable dietary practices rather than devolving into helplessness or denial [29,30,31,32,33,34,35,36,37].

However, the relationship between eco-anxiety and behavior is not linear or universally adaptive. A growing body of work documents ambivalent effects, showing that eco-anxiety may promote and inhibit action, depending on coping resources, rumination, and contextual factors [4,29,31,37,38]. Coates et al. found that climate anxiety was associated with some, but not all, proenvironmental behaviors, and that high worry could coexist with inaction in certain groups [37]. Carasso Romano and Sippori similarly distinguished personal from collective environmental behaviors, showing that ecological anxiety is more strongly related to collective action, possibly because it offers a clearer sense of efficacy and social meaning [31]. Our network model adds nuance by highlighting rumination as a bridge node that links eco-anxiety to sustainable nutrition behaviors. This pattern suggests that repetitive, focused thinking about environmental threats is a necessary—but potentially double-edged—mechanism: it can transform diffuse worry into concrete, health-related behavioral changes, yet excessive rumination might also amplify distress and impair well-being if not channeled constructively [4,29,31,32,38]. This interpretation is consistent with broader stress–vulnerability perspectives in which nutrition-related behaviors can shift under sustained psychological strain, and nutrition is often conceptualized within a wider psychophysiological vulnerability framework [39].

Our findings regarding mental well-being are broadly consistent with reviews indicating that eco-anxiety is associated with higher levels of anxiety, depressive symptoms, and stress, especially in younger populations [4,29,38]. Boluda-Verdú et al.’s systematic review showed that climate-related worry is robustly linked with adverse mental health outcomes and greater engagement in pro-environmental behavior in several studies, underscoring its ambivalent nature [38]. Similarly, Kerse and Kerse demonstrated that eco-anxiety exerts a negative direct effect on mental well-being while simultaneously promoting green buying, recycling, and low-carbon behaviors, which in turn partially buffer this negative impact [32]. Our profile structure fits this “cost–benefit” pattern: the high-eco-anxiety group appears both psychologically burdened and behaviorally engaged, whereas the lower-anxiety profiles show less distress but also weaker commitment to sustainable nutrition. This tension highlights the need for interventions that safeguard mental health while preserving the motivational value of eco-anxiety.

The network results offer additional insights into how sustainable nutrition behaviors, religious variables, and eco-anxiety are embedded within a broader psychosocial system. Sustainable nutrition nodes—particularly seasonal and local nutrition—displayed the highest centrality, suggesting that everyday food decisions structured around seasonality, locality, and minimally processed options serve as a practical “behavioral hub” connecting environmental concern, personal values, and lifestyle. This is compatible with the idea of “dietary eco-wellness,” in which environmentally conscious eating provides co-benefits for physical, planetary, and psychological health [33,34,35,36]. Religious variables occupied a more peripheral but still relevant position. Organizational and intrinsic religiosity tended to cluster together and showed modest associations with eco-anxiety profiles, particularly through the Affective-Dominant profile’s distinctively lower intrinsic religiosity. However, sensitivity analyses revealed that religiosity differences across profiles were substantially dependent on this less stable profile, with organizational and intrinsic religiosity differences becoming non-significant when excluding the Affective-Dominant profile. These findings indicate that religiosity-related interpretations should be considered preliminary and require replication in independent samples before drawing conclusions about whether faith communities and intrinsic religious commitment offer meaning, social support, and a framework for hope in the face of ecological crisis. Although the effect sizes were small, this pattern suggests that religious resources can help transform eco-anxiety into constructive engagement rather than avoidance or despair, particularly in highly religious contexts.

It should be noted that cross-construct partial correlations in the network were uniformly small (r = 0.05–0.09), indicating limited practical effect sizes despite statistical reliability with the present sample size. While sustainable nutrition nodes displayed high centrality within their own community, their connections to eco-anxiety and religiosity nodes were considerably weaker than within-construct associations. These weak cross-construct associations represent preliminary evidence of construct relationships that warrant replication before drawing strong mechanistic conclusions. The finding that constructs were more strongly connected within than between communities suggests that eco-anxiety, religiosity, and sustainable nutrition operate as relatively distinct psychological domains with only modest overlap, connected primarily through specific bridge nodes rather than through pervasive cross-domain associations.

In contrast, BMI emerged as almost isolated from the main network, with the lowest centrality indices. This is unsurprising given the cross-sectional design and the relatively young age of the sample, in which the long-term anthropometric consequences of sustainable eating patterns may not yet be fully expressed. Sustainable nutrition behaviors are likely to exert cumulative effects on body composition and cardiometabolic risk over the years rather than in the short term captured by our study. The lack of meaningful edges between BMI and either eco-anxiety or religiosity suggests that, in this demographic, weight status is shaped more by conventional determinants (e.g., physical activity, energy balance, genetics) than by climate-related worry or religious orientation. Future longitudinal studies are needed to test whether sustained engagement in ecofriendly dietary patterns predicts favorable trajectories in BMI and metabolic health [33,34,35,36,38].

Finally, the latent profile analysis underscores that eco-anxiety cannot be adequately captured by a single mean score or linear trend. The identification of four distinct profiles—including a low eco-anxiety, a moderately concerned, an affective–dominant, and a high eco-anxiety pattern—echoes recent calls to conceptualize eco-anxiety as a spectrum of experiences ranging from adaptive concern to potentially impairing distress [4,29]. The Affective-Dominant profile, while theoretically coherent as an emotionally reactive but non-ruminative pattern, showed moderate split-half replication compared to the other three profiles, suggesting that conclusions specific to this subgroup—particularly regarding religiosity—warrant independent replication before being considered robust. This typological view is clinically and policy relevant: interventions may need to be tailored differently for individuals with a high eco-anxiety profile, who might benefit from emotion regulation and burnout prevention strategies, versus those with low or moderate profiles, where the priority could be to build climate awareness and promote more sustainable behaviors. By integrating person-centered (LPA) and variable-centered (network) approaches, the present study contributes to a more differentiated understanding of how eco-anxiety, religiosity, and sustainable nutrition behaviors co-exist in a predominantly young, urban, and educated Turkish sample.

Taken together, these results suggest that sustainability-oriented eating practices co-occur with elevated eco-anxiety profiles, particularly through day-to-day decision points such as food preference formation and purchasing choices. This pattern aligns with emerging evidence that climate/eco-anxiety can accompany pro-environmental or sustainable consumption orientations, although the magnitude of associations is typically modest and directionality cannot be inferred in cross-sectional data. In other words, higher eco-anxiety may coincide with greater attentional focus on climate-related food impacts and thus more sustainable purchasing preferences, but it is equally plausible that individuals already committed to sustainable eating attend more to climate information and consequently report greater eco-anxiety. Prior studies among young adults and university samples similarly report positive associations between eco-anxiety and sustainable dietary patterns or higher adherence to sustainability-oriented dietary scores, supporting the plausibility of this co-occurrence while underscoring heterogeneity across contexts and measures [29,30,33,34,37]. The non-significant role of religiosity (with only a marginal trend for organizational participation) may reflect that religious engagement does not uniformly translate into food-related sustainability behaviors in this demographic, or that organizational religiosity is a less sensitive indicator in younger/online convenience samples; this warrants replication and more fine-grained measurement of religious moral norms directly tied to waste avoidance and stewardship in Türkiye, e.g., [13,14,15]. Finally, the lack of BMI differentiation is consistent with BMI’s peripheral role in the network and its limited sensitivity in young samples with self-reported anthropometrics.

The modest discriminative power observed in both CDA and multinomial logistic regression analyses—with classification accuracy not exceeding base rates—suggests that while eco-anxiety profiles differ systematically in sustainable nutrition behaviors at the group level, these differences are insufficient for reliable individual-level classification. Several additional limitations warrant consideration. First, the study relied on online convenience recruitment via social media; accordingly, the sample was predominantly young, female, single, highly educated, and largely composed of students. This composition increases the likelihood of selection bias (e.g., overrepresentation of digitally engaged individuals and those more interested in climate-related topics) and limits generalizability to older adults, individuals with lower educational attainment, rural populations, or groups with more restricted access to sustainable food options. Different sociodemographic and cultural contexts may yield different eco-anxiety profiles and different patterns linking religiosity and sustainable nutrition. Second, while we aimed for transparency, no prospective stage-wise attrition log was retained. To address this, we reconstructed a flow of exclusions from the raw export and data-cleaning flags: 1133 entries were recorded; 1 duplicate was removed; 15 entries with missing key variables were excluded; and 12 entries were removed due to implausible anthropometric values, yielding a final analytic sample of 1105 participants. Nevertheless, the absence of a prospective attrition log limits our ability to evaluate potential non-random dropout processes (e.g., whether individuals with higher distress were more likely to discontinue), which may further contribute to selection effects. Third, the survey did not include embedded attention checks or prespecified time-based quality screens at deployment. Although we implemented pragmatic controls (duplicate/IP/device checks, completeness screening for key indicators, and anthropometric plausibility screening), the lack of formal attention/time screens may increase measurement noise (e.g., satisficing or random responding), potentially attenuating associations and affecting profile separation and network edge estimates. Thus, small effect sizes and weak cross-construct associations should be interpreted cautiously, as they may reflect both true small effects and residual response-quality variability. Fourth, the cross-sectional design precludes causal inference, and the weak cross-construct associations observed in the network analysis do not permit conclusions about temporal ordering or directional pathways. Longitudinal or experimental designs are needed to clarify whether eco-anxiety precedes sustainable nutrition behaviors or whether engagement in sustainability-related practices shapes climate-related emotions over time. Fifth, all measures were self-reported, including height and weight used to compute BMI, which are prone to systematic reporting errors; religiosity and sustainability-related behaviors may also be influenced by social desirability. Objective measures (e.g., clinician-measured anthropometry, waist circumference or WHtR, body composition, cardiometabolic biomarkers, 24 h dietary recalls, purchasing records, or carbon footprint estimates) would strengthen future work and may reveal associations not detectable in this sample. The lack of between-profile differences in BMI may therefore reflect measurement error and restricted variability in a relatively young sample. Sixth, both LPA and network modeling depend on modeling choices and the set of included variables. Unmeasured constructs (e.g., climate activism, environmental knowledge, health anxiety, disordered eating symptoms, or broader coping styles) could alter the profile configuration and network structure. Finally, the study was conducted within a single national and religious context; cross-cultural comparative research is needed to test whether similar profiles and network structures emerge in more secular societies or in settings where sustainable foods are less affordable or accessible. Several design choices may also constrain external validity. Excluding respondents who self-reported diagnosed psychiatric or eating disorders reduces potential clinical confounding but may selectively remove individuals for whom eco-anxiety is most impairing. Likewise, excluding participants following a special diet under dietitian supervision may underrepresent individuals already engaged in strong health- or ethics-driven dietary patterns, potentially biasing associations between eco-anxiety and sustainable nutrition behaviors. Future studies should replicate these analyses in more inclusive samples and formally test sensitivity to alternative inclusion criteria.

## 5. Conclusions

In this community sample of Turkish adults, eco-anxiety, religiosity, and sustainable nutrition behaviors showed an interrelated pattern, although the network results indicate that the most robust structure was primarily within domains, with only modest cross-domain connectivity. Individuals reporting higher eco-anxiety also reported higher engagement in sustainable eating behaviors; however, given the cross-sectional design, these findings should be interpreted as associations rather than evidence of directionality. Sustainable nutrition behaviors appeared relatively central in the multivariate network, whereas BMI was largely peripheral, suggesting that body size was not a key connector among the constructs examined.

Overall, the profile and network structures are consistent with the view that eco-anxiety is not uniformly pathological, but may co-occur with value-consistent behaviors in some individuals and contexts. Religiosity—particularly intrinsic and organizational dimensions—showed preliminary associations with eco-anxiety profiles, though sensitivity analyses indicated these findings were largely dependent on the less stable Affective-Dominant profile and require replication. At the same time, eco-anxiety was also linked to greater emotional burden, underscoring that such distress may carry psychological costs alongside its association with sustainability-oriented behavior.

These preliminary findings generate hypotheses for future research using longitudinal and cross-cultural designs with objective indicators of diet and environmental impact. Should similar associations replicate, subsequent work could explore interventions combining evidence-based emotion regulation approaches with culturally appropriate, value-based (and where relevant, faith-informed) frameworks for sustainable nutrition and climate-related well-being.

## Figures and Tables

**Table 1:** Sociodemographic Characteristics Across Eco-anxiety Profiles.

Variable	Category	High (*n* = 132)	Moderate (*n* = 606)	Affective-Dominant (*n* = 92)	Low (*n* = 275)	χ^2^	*p*	Effect Size
**Age (years),** Q_2_ (Q_1_–Q_3_) [Mean (SD)]	—	22 (21–24) [24.21 (6.17)]	22 (21–26) [25.64 (8.58)]	23 (21–25) [25.38 (7.87)]	23 (21–28.2) 26.87 (9.02)	27.00	0.026	0.006
**Age Group,** *n* (%)	18–24	101 (76.5)	424 (70.0)	67 (72.8)	172 (62.5)	9.75	0.021	0.094
	25+	31 (23.5)	182 (30.0)	25 (27.2)	103 (37.5)			
**Sex,** *n* (%)	Male	33 (25.0)	178 (29.4)	18 (19.6)	110 (40.0)	19.70	<0.001	0.131
	Female	99 (75.0)	428 (70.6)	74 (80.4)	165 (60.0)			
**Marital Status,** *n* (%)	Single	114 (86.4)	479 (79.0)	75 (81.5)	212 (77.1)	5.40	0.163	0.068
	Married	18 (13.6)	127 (21.0)	17 (18.5)	63 (22.9)			
**Education,** *n* (%)	<High school	2 (1.5)	23 (3.8)	2 (2.2)	12 (4.4)	57.00	0.362	0.055
	High school	12 (9.1)	73 (12.0)	12 (13.0)	42 (15.3)			
	University	118 (89.4)	510 (84.2)	78 (84.8)	221 (80.4)			
**Religious Affiliation,** *n* (%)	Non-Muslim	15 (11.4)	47 (7.8)	15 (16.3)	26 (9.5)	7.20	0.052	0.084
	Muslim	117 (88.6)	559 (92.2)	77 (83.7)	249 (90.5)			

Values represent *n* (%) for categorical variables and M (SD) for continuous variables. Effect size = Cramer’s V for chi-square tests; η^2^ for Kruskal–Wallis test (Age).

**Table 2:** Comparison of Predictive Variables Across Eco-anxiety Profiles.

	Eco-Anxiety Profiles						
Variables	High (*n* = 132) Q_2_ (Q_1_–Q_3_)	Moderate (*n* = 606) Q_2_ (Q_1_–Q_3_)	Affective-Dominant (*n* = 92) Q_2_ (Q_1_–Q_3_)	Low (*n* = 275) Q_2_ (Q_1_–Q_3_)	χ^2^_(3)_	*p*	$\eta_{H}^{2}$
**Religiosity (DUREL)**							
Organizational Religiosity	2.5 (1–3)	3 (1–4)	2 (1–3)	2.85 (1.56)	11.34	0.010 *	0.008
Nonorganizational Religiosity	2 (1–3.25)	2 (1–4)	2 (1–3)	2.43 (1.65)	3.01	0.391	-
Intrinsic Religiosity	4 (3–4.33)	4 (3.33–4.67)	3.33 (2.67–4)	3.71 (1.04)	12.52	0.006 **	0.009
**Sustainable Nutrition Behaviors**							
Food Preference	18.5 (15–23)	18 (14–21)	18 (14–20)	16.12 (5.17)	17.22	<0.001 ***	0.013
Food Waste Reduction	33 (28–37)	33 (27–36)	32 (27–36)	30.07 (7.93)	5.42	0.144	-
Seasonal & Local Nutrition	30 (25–33)	28 (24–32)	29 (24–32)	26.34 (7.23)	11.62	0.009 **	0.008
Food Purchasing	22 (18–26)	20 (16–24)	19 (16–23)	18.71 (5.60)	18.68	<0.001 ***	0.014
**Anthropometric**							
BMI (kg/m^2^)	22.27 (19.56–25.53)	22.97 (20.54–25.77)	22.27 (19.47–25.76)	23.74 (4.56)	4.52	0.211	-

Values represent Median (Q_1_–Q_3_). $\eta_{H}^{2}$ = epsilon-squared effect size for Kruskal–Wallis test. Wilks’ λ = 0.943, F_(24,3173.5)_ = 2.68, *p* < 0.001; Function 1: Canonical R^2^ = 0.032, Eigenvalue = 0.033, Variance = 56.2%, *p* < 0.001; Function 2: Canonical R^2^ = 0.024, Eigenvalue = 0.024, Variance = 40.9%, *p* = 0.014; Function 3: Canonical R^2^ = 0.002, Eigenvalue = 0.002, Variance = 2.9%, *p* = 0.929; Classification Accuracy = 54.9%; * *p* < 0.05; ** *p* < 0.01; *** *p* < 0.001.

**Table 3:** Multinomial Logistic Regression Predicting Eco-Anxiety Profile Membership.

Predictor	Moderate			Affective-Dominant			High		
	OR [95% CI]	z	*p*	OR [95% CI]	z	*p*	OR [95% CI]	z	*p*
Org. Religiosity	0.96 [0.81–1.14]	−0.48	0.635	0.74 [0.55–1.01]	−1.87	0.061	0.77 [0.59–1.00]	−1.93	0.053
Non-Org. Religiosity	0.96 [0.81–1.14]	−0.43	0.667	1.16 [0.87–1.54]	0.99	0.324	0.91 [0.70–1.18]	−0.72	0.473
Intrinsic Religiosity	1.09 [0.91–1.30]	0.93	0.352	0.76 [0.58–1.00]	−1.96	0.050 *	1.07 [0.83–1.37]	0.48	0.629
Food Preference	1.25 [1.02–1.52]	2.17	0.030 *	1.12 [0.81–1.53]	0.69	0.493	1.58 [1.17–2.14]	2.97	0.003 **
Waste Reduction	1.00 [0.79–1.27]	−0.02	0.984	1.03 [0.69–1.52]	0.13	0.897	0.82 [0.57–1.18]	−1.07	0.287
Seasonal/Local	0.93 [0.72–1.21]	−0.51	0.607	1.60 [1.04–2.47]	2.15	0.032 *	0.96 [0.65–1.43]	−0.19	0.853
Food Purchasing	1.10 [0.87–1.41]	0.80	0.424	0.77 [0.53–1.14]	−1.30	0.194	1.47 [1.02–2.12]	2.04	0.041 *
BMI	0.97 [0.84–1.12]	−0.43	0.668	0.84 [0.66–1.08]	−1.33	0.183	0.92 [0.74–1.14]	−0.78	0.434

Reference category = Low eco-anxiety profile. All predictors were standardized prior to analysis. OR = odds ratio; CI = confidence interval. Model fit: Likelihood Ratio χ^2^(24) = 65.87, *p* < 0.001; McFadden R-squared = 0.026; Nagelkerke R-squared = 0.064. Classification accuracy = 54.7% (base rate = 54.7%). * *p* < 0.05; ** *p* < 0.01.

## Data Availability

The data presented in this study are available on request from the corresponding author due to ethics/privacy restrictions.

## Supplementary Materials

The following supporting information can be downloaded at: https://www.mdpi.com/article/10.3390/nu18030545/s1, File S1: Detailed Statistical Results. Section S1: Psychometric Properties of Study Instruments; Table S1: Internal Consistency Coefficients for Study Instruments; Table S2: Confirmatory Factor Analysis Fit Indices; Table S3: Model Modifications Applied During CFA; Table S4: Subscale Intercorrelation Matrix; Section S2: Latent Profile Analysis Model Selection and Profile Characteristics; Table S5: Latent Profile Analysis Model Fit Comparison; Table S6: Profile-Specific Means and Standardized Scores for Eco-Anxiety Subscales; Table S7: Measurement Invariance Across Gender; Table S8: Measurement Invariance Across Age Groups; Table S9: Profile Stability Analysis Results; Section S3: Sensitivity Analyses for Profile Stability; Table S10: Kruskal–Wallis Tests Full Sample vs. Sensitivity Sample.

## Abbreviations

The following abbreviations are used in this manuscript:

BMI	Body Mass Index
CDA	Canonical discriminant analysis
DUREL	Duke University Religion Index
EBICglasso	Extended Bayesian Information Criterion graphical LASSO
EAS	Eco-Anxiety Scale
GGM	Gaussian Graphical Model
LPA	Latent Profile Analysis
