# Supplementary material for: Eco-Anxiety Profiles, Religiosity, and Sustainable Nutrition in Turkish Adults: A Latent Profile and Network Analysis
Source: Nutrients. 2026 Feb 6;18(3):545. doi: 10.3390/nu18030545 (PMC12899786; doi:10.3390/nu18030545)
Supplement: Supplementary file 1 [file nutrients-18-00545-s001.zip › nutrients-4109681-supplementary.pdf]

Supplementary Materials

Section 1: Psychometric Properties of Study Instruments

S1.1. Internal Consistency Coefficients

Table S1 presents internal consistency coefficients for all study instruments. Cronbach's alpha and McDonald's omega total were calculated for total scales and subscales. All coefficients exceeded the recommended threshold of 0.70, indicating acceptable to excellent internal consistency.

Table S1. Internal Consistency Coefficients for Study Instruments (N = 1,105)

Scale / Subscale	Items	Alpha	Omega
**Eco-Anxiety Scale (Total)**	13	.901	.942
Affective Symptoms	4	.869	-
Rumination	3	.864	-
Behavioral Symptoms	3	.788	-
Personal Impact Anxiety	3	.852	-
**Sustainable Nutrition Behaviors Scale (Total)**	29	.958	.968
Food Preference	6	.869	-
Food Waste Reduction	9	.906	-
Seasonal and Local Nutrition	8	.914	-
Food Purchasing	6	.852	-
**Duke University Religion Index (Total)**	5	.811	.872
Organizational Religiosity	1	-	-
Non-Organizational Religiosity	1	-	-
Intrinsic Religiosity	3	.851	-

Alpha = Cronbach's alpha; Omega = McDonald's omega total. Dashes indicate coefficients not calculable for single-item subscales or not reported for subscales when omega was computed at the total scale level.

S1.2. Confirmatory Factor Analysis

Confirmatory factor analysis (CFA) was conducted using the lavaan package in R with robust maximum likelihood estimation (MLR) to account for non-normality. Initial models were evaluated and subsequently refined based on modification indices, with correlated residuals added only between items within the same subscale and with theoretically justifiable relationships (i.e., similar item content or method effects).

Table S2. Confirmatory Factor Analysis Fit Indices

Scale	χ^2 (df)	CFI	TLI	RMSEA	90% CI	SRMR
Eco-Anxiety Scale	230.05 (57)	.968	.956	.052	[.045, .060]	.048
Sustainable Nutrition	1471.65 (361)	.919	.909	.053	[.050, .056]	.056
DUREL	6.57 (3)	.998	.994	.033	[.000, .068]	.013

χ^2 = Satorra-Bentler scaled chi-square; CFI = Comparative Fit Index; TLI = Tucker-Lewis Index; RMSEA = Root Mean Square Error of Approximation; CI = Confidence Interval; SRMR = Standardized Root Mean Square Residual. Recommended cut-offs: CFI > .90, TLI > .90, RMSEA < .08, SRMR < .08.

Table S3. Model Modifications Applied During CFA

Scale	Correlated Residuals	Rationale
**Eco-Anxiety Scale** (2 modifications)	EKO1 ~~ EKO2	Both assess affective responses (fear/worry)
	EKO5 ~~ EKO6	Both assess ruminative thinking patterns
**Sustainable Nutrition** (10 modifications)	SURBES5 ~~ SURBES6	Both assess food preference decisions
	SURBES7 ~~ SURBES8	Adjacent waste reduction behaviors
	SURBES9 ~~ SURBES10	Similar food storage practices
	SURBES10 ~~ SURBES11	Related leftover management items
	SURBES12 ~~ SURBES13	Both assess portion control behaviors
	SURBES14 ~~ SURBES15	Related meal planning items
	SURBES16 ~~ SURBES17	Both assess seasonal food selection
	SURBES21 ~~ SURBES22	Similar local sourcing preferences
	SURBES24 ~~ SURBES27	Both assess purchasing considerations
	SURBES28 ~~ SURBES29	Both assess environmental purchasing
**DUREL** (2 modifications)	durel1 ~~ durel2	Both assess external religious activities
	durel3 ~~ durel4	Both assess intrinsic religious experience

Modifications were added sequentially based on modification indices (MI > 10) and theoretical justification. Only within-subscale correlated residuals with positive expected parameter change were considered.

S1.3. Subscale Intercorrelations

Table S4 presents Pearson correlations among all subscales. As expected, subscales within the same instrument showed moderate to strong correlations, while cross-instrument correlations were generally weaker. Notably, eco-anxiety subscales (particularly Rumination and Personal Impact) showed small but significant positive correlations with sustainable nutrition behaviors, whereas religiosity subscales showed near-zero correlations with eco-anxiety.

Table S4. Subscale Intercorrelation Matrix

Variable	1	2	3	4	5	6	7	8	9	10	11
1. EKO-Aff	-										
2. EKO-Rum	.48	-									
3. EKO-Beh	.53	.34	-								
4. EKO-Per	.48	.61	.49	-							
5. DUR-Org	-.08	-.02	.01	-.03	-						
6. DUR-Non	-.02	-.02	-.04	-.04	.48	-					
7. DUR-Int	-.01	.03	-.05	.01	.48	.45	-				
8. SUR-Prf	.10	.22	.03	.18	.08	.12	.21	-			
9. SUR-Wst	.09	.15	-.01	.12	.13	.20	.24	.60	-		
10. SUR-Sea	.12	.17	.00	.13	.11	.17	.24	.66	.77	-	
11. SUR-Pur	.09	.20	.02	.19	.09	.15	.23	.63	.72	.77	-

EKO = Eco-Anxiety Scale (Aff = Affective, Rum = Rumination, Beh = Behavioral, Per = Personal Impact); DUR = DUREL (Org = Organizational, Non = Non-Organizational, Int = Intrinsic); SUR = Sustainable Nutrition (Prf = Food Preference, Wst = Waste Reduction, Sea = Seasonal/Local, Pur = Purchasing). Correlations |r| > .06 are significant at p < .05; |r| > .08 at p < .01; |r| > .10 at p < .001.

S1.4. Summary of Psychometric Evaluation

All three instruments demonstrated satisfactory psychometric properties in the present sample. The Eco-Anxiety Scale showed excellent internal consistency (alpha = .90, omega = .94) and good model fit (CFI = .97, RMSEA = .05). The Sustainable Nutrition Behaviors Scale exhibited excellent reliability (alpha = .96, omega = .97) and acceptable fit after incorporating theoretically justified correlated residuals (CFI = .92, RMSEA = .05). The DUREL showed good internal consistency (alpha = .81, omega = .87) and excellent model fit (CFI = .998, RMSEA = .03). The pattern of subscale intercorrelations supported the discriminant validity of the constructs, with within-scale correlations consistently stronger than between-scale correlations. These findings support the use of these instruments for the subsequent latent profile and network analyses.

Section 2: Latent Profile Analysis: Model Selection and Profile Characteristics

S2.1. Model Fit Comparison

Latent profile analysis was conducted to identify distinct subgroups based on the four eco-anxiety subscales (Affective Symptoms, Rumination, Behavioral Symptoms, and Personal Impact Anxiety). Models specifying one through six latent profiles were estimated using the tidyLPA package in R with mclust estimation. All models used equal variances and zero covariances (Model 1 specification), as alternative specifications either failed to converge or yielded poor classification quality.

Table S5. Latent Profile Analysis Model Fit Comparison

Profiles	AIC	BIC	aBIC	Entropy	AvePP Min	AvePP Max	Smallest Class (%)	BLRT p
1	20167.22	20207.44	20182.21	1.000	1.000	1.000	100.0	-
2	19228.68	19293.96	19252.03	0.817	0.890	0.969	24.4	-
3	18953.57	19043.90	18985.28	0.748	0.840	0.910	12.7	-
4*	18825.04	18940.43	18865.11	0.773	0.715	0.911	8.4	-
5	18526.15	18666.60	18574.58	0.931	0.702	0.983	3.4	-
6	18607.87	18773.37	18664.66	0.820	0.691	0.934	3.1	-

*Note. AIC = Akaike Information Criterion; BIC = Bayesian Information Criterion; aBIC = sample-adjusted BIC; AvePP = Average Posterior Probability. *Selected solution. The 4-profile solution was selected based on entropy, classification quality, minimum class size (>5%), and interpretability criteria.*

S2.2. Profile-Specific Means and Standardized Scores

Table S6 presents the estimated means, standard errors, and standardized z-scores for each eco-anxiety subscale across the four identified profiles. Z-scores were computed as (profile mean - grand mean) / grand SD to facilitate comparison across subscales with different metrics.

Table S6. Profile-Specific Means and Standardized Scores for Eco-Anxiety Subscales

Subscale	High *n=132 (11.9%)*		Moderate *n=606 (54.8%)*		Affective-Dominant *n=92 (8.3%)*		Low *n=275 (24.9%)*	
	M (SE)	z	M (SE)	z	M (SE)	z	M (SE)	z
Affective Symptoms	12.80 (0.44)	1.39	8.43 (0.12)	-0.14	12.74 (0.52)	1.37	6.43 (0.25)	-0.84
Rumination	9.26 (0.52)	1.60	6.10 (0.13)	0.15	5.20 (0.57)	-0.27	3.74 (0.11)	-0.94
Behavioral Symptoms	9.03 (0.42)	1.18	6.12 (0.09)	-0.08	8.40 (0.43)	0.91	4.65 (0.18)	-0.72
Personal Impact Anxiety	9.43 (0.56)	1.53	6.33 (0.12)	0.11	6.34 (0.87)	0.11	4.00 (0.17)	-0.96
AvePP	.894		.875		.817		.875	

M = estimated profile mean; SE = standard error; z = standardized score; AvePP = average posterior probability of profile membership. Model specification: Equal variances, zero covariances. Entropy = 0.773.

S2.3. Classification Diagnostics

Classification quality was evaluated through multiple indicators. Average posterior probabilities for most likely class membership ranged from 0.817 (Affective-Dominant) to 0.894 (High), all exceeding the recommended threshold of 0.70. The entropy value of 0.773 indicates acceptable classification precision, with values above 0.70 generally considered adequate for person-centered analyses. All four profiles exceeded the recommended 5% minimum class size, with proportions ranging from 8.4% (Affective-Dominant) to 54.8% (Moderate).

S2.4. Measurement Invariance

Measurement invariance of the Eco-Anxiety Scale was tested across gender and age groups using multigroup confirmatory factor analysis with robust maximum likelihood estimation (MLR). Four levels of invariance were evaluated: configural (same factor structure), metric (equal factor loadings), scalar (equal intercepts), and strict (equal residual variances). Chen's 2007 criteria's model comparison used changes in CFI and RMSEA, with |ΔCFI| < .010 and |ΔRMSEA| < .015 indicating invariance.

Table S7. Measurement Invariance Across Gender (Male n = 339; Female n = 766)

Model	χ^2	df	CFI	RMSEA	SRMR	ΔCFI	ΔRMSEA	ΔSRMR
Configural	328.14	118	.962	.057	.049	-	-	-
Metric	341.58	127	.961	.055	.050	-.001	-.002	.001
Scalar	381.44	136	.955	.057	.051	-.006	.002	.001
Strict	391.97	149	.956	.054	.051	.001	-.003	.000

χ^2 = Satorra-Bentler scaled chi-square; CFI = Comparative Fit Index; RMSEA = Root Mean Square Error of Approximation; SRMR = Standardized Root Mean Square Residual; ΔCFI = change in CFI; ΔRMSEA = change in RMSEA; ΔSRMR = change in SRMR. Recommended thresholds for invariance: |ΔCFI| < .010, |ΔRMSEA| < .015, |ΔSRMR| < .03. All values fall within acceptable thresholds, supporting full strict invariance across gender.

Table S8. Measurement Invariance Across Age Groups (18-24 years n = 764; 25+ years n = 341)

Model	χ^2	df	CFI	RMSEA	SRMR	ΔCFI	ΔRMSEA	ΔSRMR
Configural	329.11	118	.961	.057	.049	-	-	-
Metric	339.12	127	.961	.055	.050	.000	-.002	.001
Scalar	360.06	136	.959	.055	.050	-.002	.000	.000
Strict	379.53	149	.957	.053	.051	-.002	-.002	.001

Note. Abbreviations as in Table S7. Recommended thresholds: |ΔCFI| < .010, |ΔRMSEA| < .015, |ΔSRMR| < .030. All values fall within acceptable thresholds, supporting full strict invariance across age groups.

S2.5. Profile Stability Analysis

To evaluate the stability of the four-profile solution, we conducted bootstrap resampling (1,000 iterations) and split-half cross-validation analyses.

Table S9. Profile Stability Analysis Results

Analysis	Metric	Value
**Bootstrap (n = 1,000)**	4-profile recovery rate	100%
**Split-half cross-validation**	Half 1-sample size	n = 552
	Half 2-sample size	n = 553
**Profile pattern correlations**	High	r = .929
	Low	r = .959
	Affective-Dominant	r = .585
	Moderate	r = .985
	Mean pattern correlation	r = .865

Note. Profile pattern correlations represent the correlation between profile centroids (means across four eco-anxiety subscales) estimated independently in each half of the sample, after optimal alignment. Values > .80 indicate excellent replication; values .60-.80 indicate acceptable replication.

The 100% bootstrap recovery rate indicates that the four-profile structure was consistently identified across resamples. Split-half cross-validation revealed that three of four profiles (High, Low, Moderate) showed excellent pattern replication (r > .90), while the Affective-Dominant profile showed moderate replication (r = .585), likely reflecting its smaller size and more distinctive configuration. The mean pattern correlation of .865 supports the overall stability of the profile solution.

Section 3: Sensitivity Analyses for Profile Stability

S3.1. Rationale

Given the moderate split-half replication of the Affective-Dominant profile (r = .585) and its relatively small size (n = 92, 8.3%), sensitivity analyses were conducted to evaluate whether key findings were contingent upon this profile. All primary analyses were repeated excluding participants classified into the Affective-Dominant profile (sensitivity sample n = 1,012).

S3.2. Comparison of Profile Differences

Table S10 presents Kruskal-Wallis test results comparing the full sample (four profiles) with the sensitivity sample (three profiles: High, Moderate, Low).

Table S10. Kruskal-Wallis Tests: Full Sample vs. Sensitivity Sample

Variable	χ2 (Full)	η^2	p	χ2 (Sensitivity)	η^2	p
**Sustainable Nutrition**						
Food Preference	17.74	.013	**< .001**	17.57	.015	**< .001**
Food Waste Reduction	5.55	.002	.136	5.47	.003	.065
Seasonal & Local	11.33	.008	**.010**	10.12	.008	**.006**
Food Purchasing	18.59	.014	**< .001**	18.03	.016	**< .001**
**Religiosity (DUREL)**						
Organizational	12.01	.008	**.007**	5.46	.003	.065
Non-Organizational	2.77	< .001	.428	2.19	< .001	.334
Intrinsic	13.52	.010	**.004**	0.21	< .001	.902
**BMI**	4.64	.002	.200	2.93	.001	.231

Note. Full sample: N = 1,105 (4 profiles); Sensitivity sample: n = 1,012 (3 profiles, excluding Affective-Dominant). Bold p-values indicate statistical significance (p < .05). η^2 = eta-squared effect size.

S3.3. Key Findings

Sustainable nutrition findings remained robust. All three sustainable nutrition subscales that were significant in the full sample (Food Preference, Seasonal & Local, Food Purchasing) remained significant in the sensitivity analysis with comparable or slightly larger effect sizes. The monotonic pattern of higher eco-anxiety profiles showing greater sustainable nutrition behaviors (High > Moderate > Low) was preserved.

Religiosity findings were profile-dependent. The significant differences in Organizational Religiosity (p = .007) and Intrinsic Religiosity (p = .004) observed in the full sample became non-significant when excluding the Affective-Dominant profile (p = .065 and p = .902, respectively). This suggests that religiosity-related interpretations are substantially influenced by the Affective-Dominant profile, which showed distinctively lower intrinsic religiosity (Mean = 3.41) compared to other profiles (Mean = 3.71-3.81).

S3.4. Discriminant Analysis Sensitivity

MANOVA results in the sensitivity sample remained significant (Wilks' Lambda = 0.962, $F_{(16, 2004)}$ = 2.46, p = .001), though with reduced effect. The first discriminant function (sustainable nutrition dimension) accounted for 92.0% of between-group variance in the sensitivity sample compared to 56.2% in the full sample, indicating that sustainable nutrition variables become more dominant discriminators when the Affective-Dominant profile is excluded. Classification accuracy (59.7%) equaled the base rate of the largest class (Moderate, 59.7%), consistent with the modest effect sizes observed.

S3.5. Summary and Implications

The sensitivity analyses confirm that the primary conclusion regarding the positive association between eco-anxiety and sustainable nutrition behaviors does not depend on the Affective-Dominant profile and is robust across analytical approaches. However, findings regarding religiosity differences across profiles should be interpreted with caution, as these effects appear to be driven primarily by the Affective-Dominant profile. Given the moderate replication stability of this profile ($r = .585$), religiosity-related interpretations should be considered preliminary and require replication in independent samples.
